# Defining criteria for broadly neutralizing HIV antibodies

**DOI:** 10.3389/fimmu.2025.1624020

**Published:** 2025-07-17

**Authors:** Elizabeth-Sharon David-Fung, Katherine A. Belobrajdic, Jennifer P. Macke, Corey R. Quackenbush, Kumkum Ganguly, Jennifer L. Mamrosh

**Affiliations:** Theoretical Biology and Biophysics, Los Alamos National Laboratory, Los Alamos, NM, United States

**Keywords:** HIV, broadly neutralizing antibodies, BNAB, antibody breadth, potency

## Abstract

Over the course of a few years, a small percentage of individuals with HIV-1 develop broadly neutralizing antibodies (bnAbs) capable of neutralizing diverse viruses. Although hundreds of antibodies with neutralizing activity against heterologous viruses have been referred to as bnAbs, there is no universally accepted numerical definition of a bnAb. Here, we will review important elements of HIV neutralizing antibodies and proposed definitions of bnAbs, as well as introduce a web-based tool, CAByN (Choose Antibodies by Neutralization), allowing users to identify antibodies meeting their numerical definitions of a bnAb from data in the Los Alamos HIV Databases CATNAP (Compile, Analyze and Tally NAb Panels) antibody neutralization database. Biological findings from use of CAByN are also presented here, including differential neutralizing activity for certain antibodies across viral clades, and identification of antibodies with suspected incomplete neutralization. Website address: http://hiv.lanl.gov/content/sequence/CABYN/CABYN.html.

## 1 Introduction

Although development of mature antibodies lags behind T cell responses to initial HIV-1 infection (1), these antibodies have the potential to slow or stop HIV progression. To do so, antibodies need to develop the capability to neutralize HIV strains beyond that which they were initially exposed to, given the rapid mutation rate of HIV. Antibodies neutralizing diverse strains of HIV are termed “broadly neutralizing antibodies” (bnAbs) and generally target the HIV Envelope (Env) protein, which exists as a heavily glycosylated trimer. Only approximately 1% of individuals with HIV develop antibodies with the neutralization potency and breadth likely needed to control HIV infection (2).

Antibody potency is an important concern for therapeutically administered bnAbs, as less antibody will need to be administered for more potent bnAbs, and efforts have been made to optimize bnAb potency for this purpose (3). Potency *in vivo* is similarly important given that antibodies compete with other antibodies of various potencies targeting similar epitopes (4). More potent HIV antibodies are also more likely to target closed Env trimers and cause less structural changes upon binding (5).

Additionally, bnAbs must have sufficient breadth to combat strains of HIV in latently infected cells as well as newly evolving HIV strains. Administration of potent bnAbs to individuals in the AMP trials demonstrated that, while antibody administration made individuals less likely to acquire HIV for infecting strains sensitive to the antibody, it could not prevent infection by more divergent resistant strains (6). In humans that produce bnAbs, the process of antibody development typically takes a few years (1) and occurs iteratively as HIV evolves, necessitating antibody evolution. Exposure to diverse strains of HIV is an important element of this process. This is supported by the higher likelihood of bnAb production by individuals dually infected with discordant strains of HIV (7). Many current HIV vaccine strategies aim to emulate this process by immunizing sequentially with distinct immunogens designed to promote the development of antibody breadth (8).

**Figure 1:**
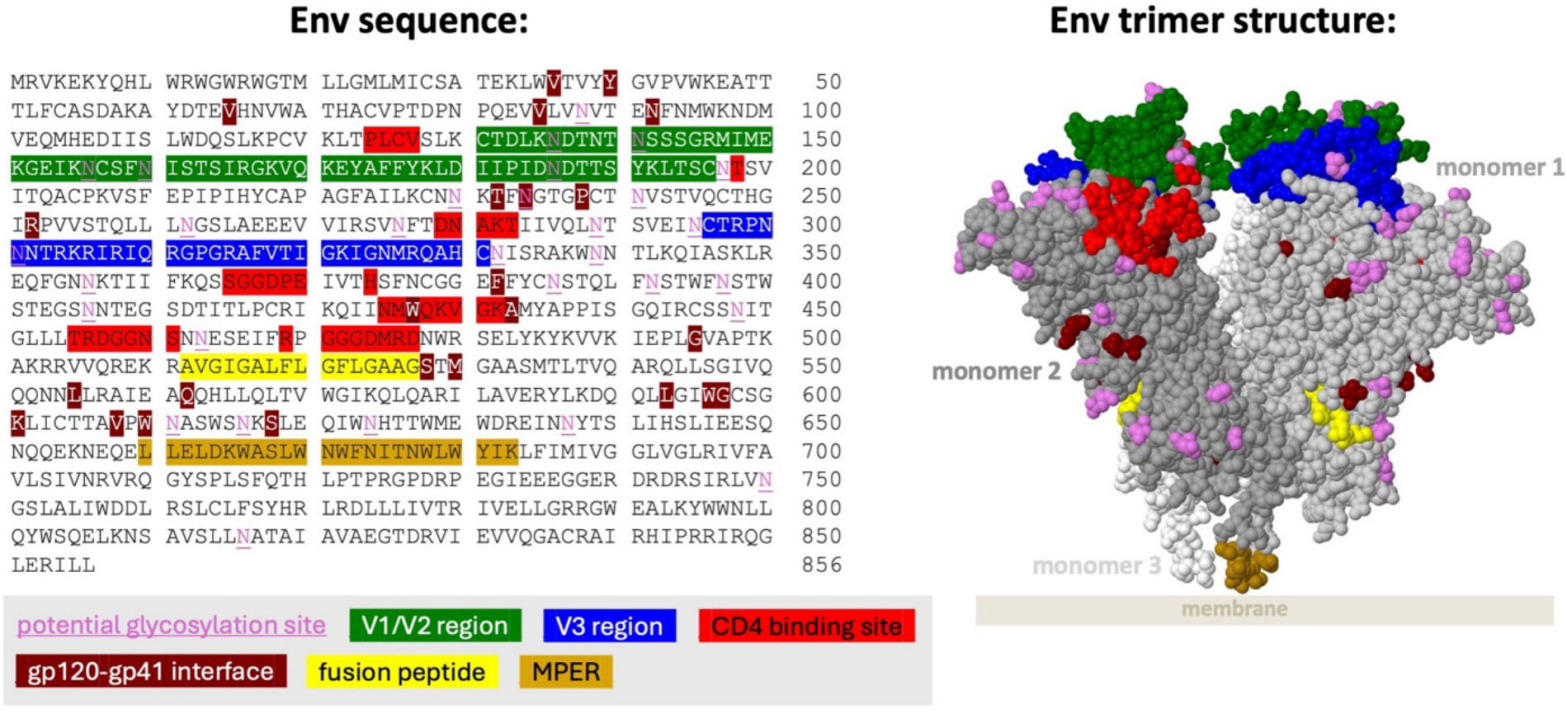
Regions of HIV-1 Env frequently targeted by bnAbs. Env HXB2 sequence amino acid residues 1–511 are gp120 and residues 512–856 are gp41. Regions are only colored on monomers 1 and 2. Note that glycans in the V1/V2 and V3 regions are preferentially targeted by many bnAbs. Regions are shown on the space filled structure of the HIV-1 Envelope protein in native membrane (PDB 7SKA), in which the transmembrane domain and part of the MPER (membrane proximal external region) domain were removed. The silent face region is not shown as it has only been approximately defined (22). Coordinates for each region excluding the silent face are available at hiv.lanl.gov.

Antibody development against HIV is highly constrained by the dense glycan shield of the Env protein. Therefore, most bnAbs target only several regions of the Env protein (**Figure 1**). Antibody characteristics differ between the regions targeted (**Table 1**), giving insight into how antibodies evolve to neutralize HIV. For example, antibodies targeting the V1/V2 region often have unusually long CDRH3 loops, which are helpful in penetrating the glycan shield in this rapidly evolving region of Env.

**Table 1:** Characteristics of antibodies targeting different regions of Env.

Antibody binding region	% Abs	% SHM	CDRH3 length	%Auto/polyreactive	Entropy
V3	27	13 ± 0.5	20 ± 0.3	16	0.6 ± 0.1
CD4bs	25	17 ± 1.2	16 ± 0.3	16	0.5 ± 0.1
V1/V2	18	13 ± 0.5	27 ± 0.8	8	1.3 ± 0.1
glycan	12	18 ± 0.9	23 ± 0.9	36	1.0 ± 0.1
fusion peptide	9	15 ± 1.3	14 ± 0.9	2	0.4 ± 0.1
MPER	5	13 ± 1.0	20 ± 0.6	71	0.4 ± 0.1
gp120-gp41 interface	1	25 ± 1.9	16 ± 1.7	5	0.4 ± 0.1
silent face	1	25 ± 3.3	21 ± 1.3	0	ND

Only natural monoclonal antibodies were used for this analysis, which excludes polyclonal mixtures, UCA/intermediates, and germline antibodies. When antibodies had more than one value, the highest value was used. % of antibodies is the percentage of natural monoclonal antibodies with the binding region in LANL’s CATNAP antibody neutralization database (11). Note that some antibodies can bind multiple antibody binding regions. % somatic hypermutation (SHM) is the average ± standard error of heavy chain nucleic acid SHM percentages for antibodies in LANL’s CATNAP database. CDRH3 length is the average ± standard error of CDRH3 lengths for antibodies in LANL’s CATNAP database. % poly/autoreactive is the percent of antibodies with this binding region in LANL’s HIV Immunology Database with an assigned “antibody polyreactivity” and/or “autoreactivity” keyword. Entropy of binding region is the average ± standard error of entropy scores calculated for Env amino acid positions in LANL’s 2022 filtered web alignment. HXB2 and BG505 SOSIP.664 glycosites were used for the glycan binding region. ND, not determined. Values >20% higher than the average across all antibodies are colored.

Despite the importance of bnAbs in HIV control and intense research focus on their elicitation, there is not a universally accepted definition of a bnAb beyond the subjective classification of it as being both relatively potent and broadly neutralizing. Custom definitions of what constitutes a bnAb may be appropriate when considering differing research and treatment aims; for example, an antibody with high breadth across a single HIV clade may be effective prophylactic treatment for individuals expected to just be exposed to this clade. Early work on antibodies targeting Env revealed that a subset of antibodies was able to neutralize most “Tier 2” and some “Tier 3” viruses (in which the Env trimer is in a closed state), in addition to the more easily neutralized “Tier 1” viruses. bnAbs elicited by HIV vaccine trials were thus expected to broadly neutralize Tier 2 viruses (9). In more recent years, more precise definitions of bnAbs have been proposed. “Elite” neutralization activity has been defined as the ability to neutralize “on average, more than one pseudovirus at an IC(50) titer of 300 within a clade group and across at least four clade groups” (2). More recently, bnAbs were proposed to be second generation bnAbs with greater than 30% breadth across 118 multi-clade panel viruses and potency ≤ 3.6 µg/ml. A second category of “elite bnAbs” was defined as those with greater than 68% breadth across 118 multi-clade panel viruses and potency < 0.06 µg/ml (10).

Given this uncertainty in which criteria are best used to identify bnAbs, our group has developed a web-based tool, CAByN (Choose Antibodies by Neutralization), allowing users to define criteria for bnAbs and obtain a list of antibodies meeting their criteria. CAByN analyzes data in the Los Alamos HIV Database CATNAP (Compile, Analyze and Tally NAb Panels) antibody neutralization database (11). CAByN has default criteria for bnAb searches, which will generate a list of bnAbs or bnAb-like antibodies, but we expect that many users will use their own criteria. Ultimately, we hope that usage of CAByN will encourage the definition of numerical criteria for bnAbs.

## 2 Method

Our Choose Antibodies by Neutralization (CAByN) tool analyzes antibody neutralization data in the Los Alamos CATNAP neutralization database (Compile, Analyze and Tally NAb Panels) (11). CATNAP is a public repository of antibody neutralization data collected from published reports and, occasionally, personal communications. CATNAP also contains tools for analyzing this data. For example, users can select specific antibodies and viruses, and CATNAP’s data analysis tool will show users aligned virus sequences alongside a geometric mean IC50/IC80 value for all assays performed for the virus/antibody combination. CATNAP allows users to identify viral sequence features associated with unusually good or poor neutralization activity, and it can also be used to help researchers identify antibodies likely to have neutralization activity against viruses resistant to certain antibodies. CAByN does not offer the ability to specifically assess an antibody’s neutralization properties against a specific virus; instead, its strength lies in allowing users, upon specifying classes of antibodies and/or viruses of interest, to identify the top neutralizing antibodies based on their neutralization potency and breadth. For example, CATNAP would allow a user to investigate whether the N332 glycosite is important for the neutralization activity of the 10–1074 antibody, whereas CAByN would allow users to find all antibodies meeting specified criteria for neutralization (a CAByN search with default settings finds that 10–1074 is ranked 60^th^ of 162 antibodies meeting search criteria in terms of mean IC50 of selected data, and 10–1074 is ranked 70^th^ for neutralization breadth). We expect that CAByN and CATNAP can be used in complementary manners, most likely with CATNAP used after a CAByN search to address more specific research questions.

Additionally, CAByN incorporates information from the Los Alamos HIV Molecular Immunology Database.

**Figure 2:**
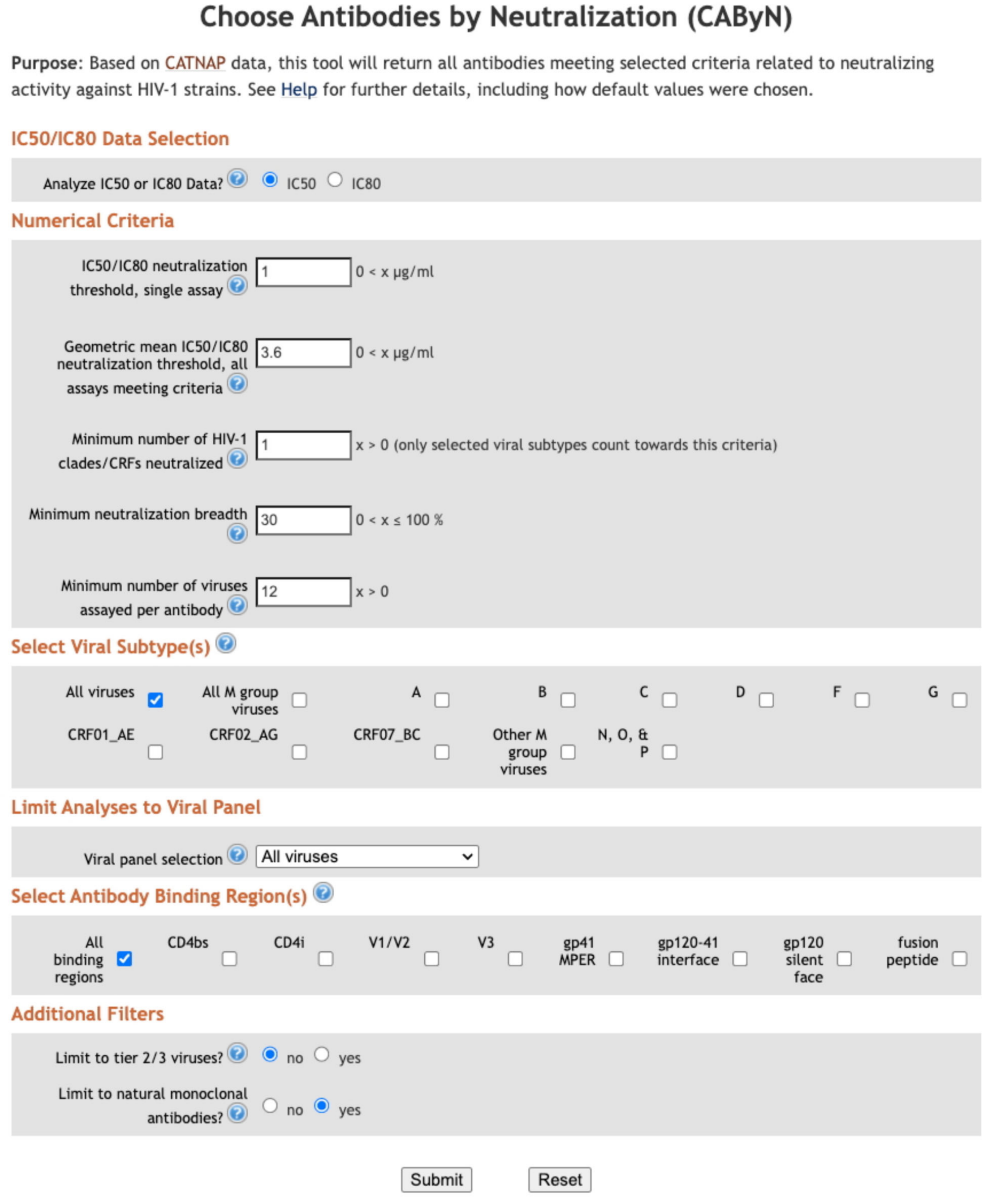
CAByN input page.

**Figure 3:**
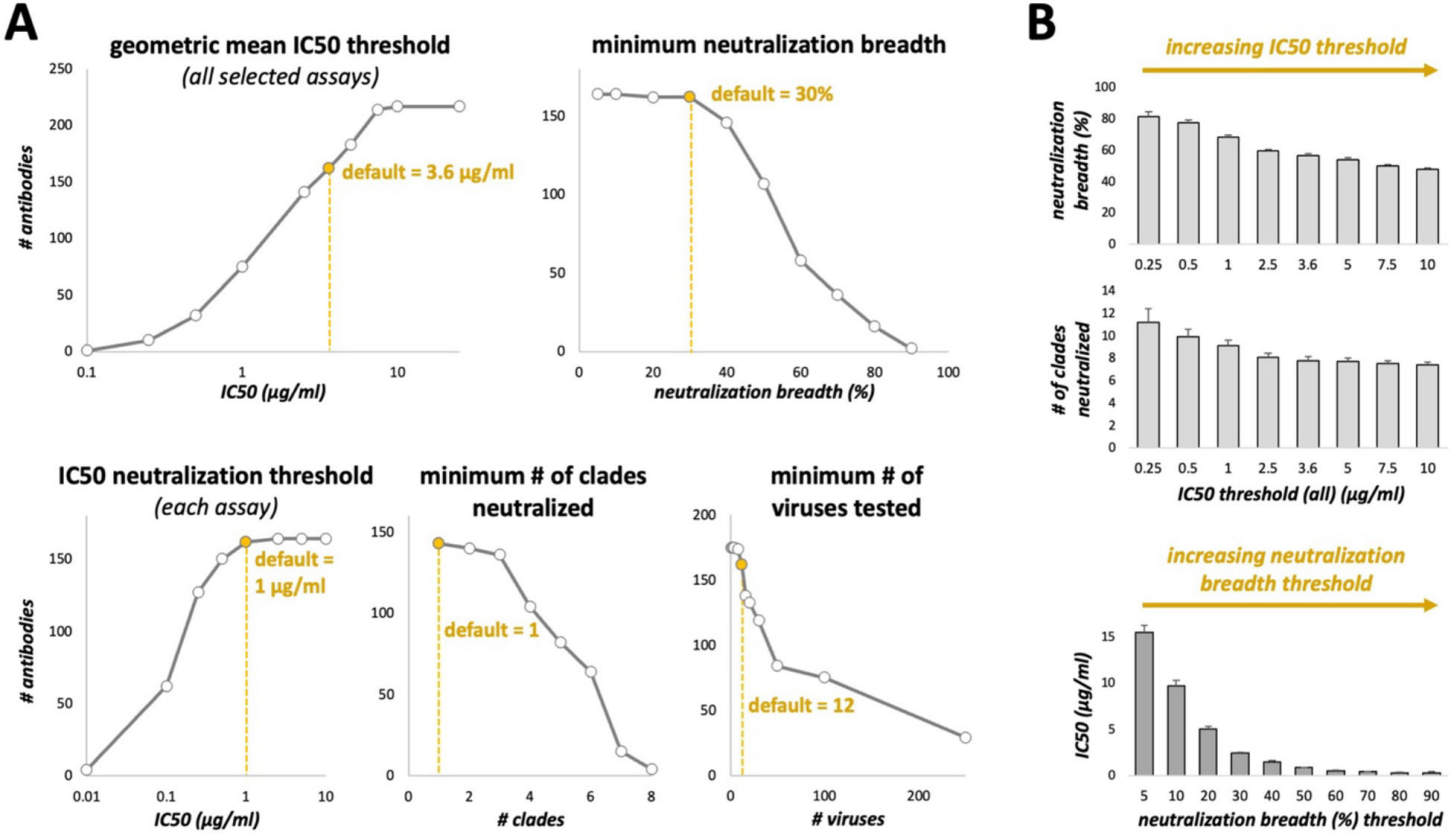
Effect of changing CAByN default settings on the number of antibodies returned. **(A)** CAByN default settings were used except for the setting listed above each graph. IC50 values were analyzed. Only the following clades were selected for the “minimum # of clades neutralized” comparison: A, B, C, D, F, G, CRF01_AE, and N/O/P. **(B)** CAByN default settings were used except that for the top two graphs, IC50 threshold (all) was set to that indicated on the x-axis, and minimum neutralization breadth was set to 0% (top graph). For the bottom graph, neutralization breadth threshold was set to that indicated on the x-axis, and IC50 threshold (all) was set to 100 µg/ml. The y-axis for all graphs indicates the average value ± SEM for all antibodies returned by that search. For example, the leftmost bar in the top graph represents the average neutralization breadth for antibodies returned by a CAByN search with default settings except that the IC50 threshold (all) was set to 0.25 µg/ml.

**Figure 4:**
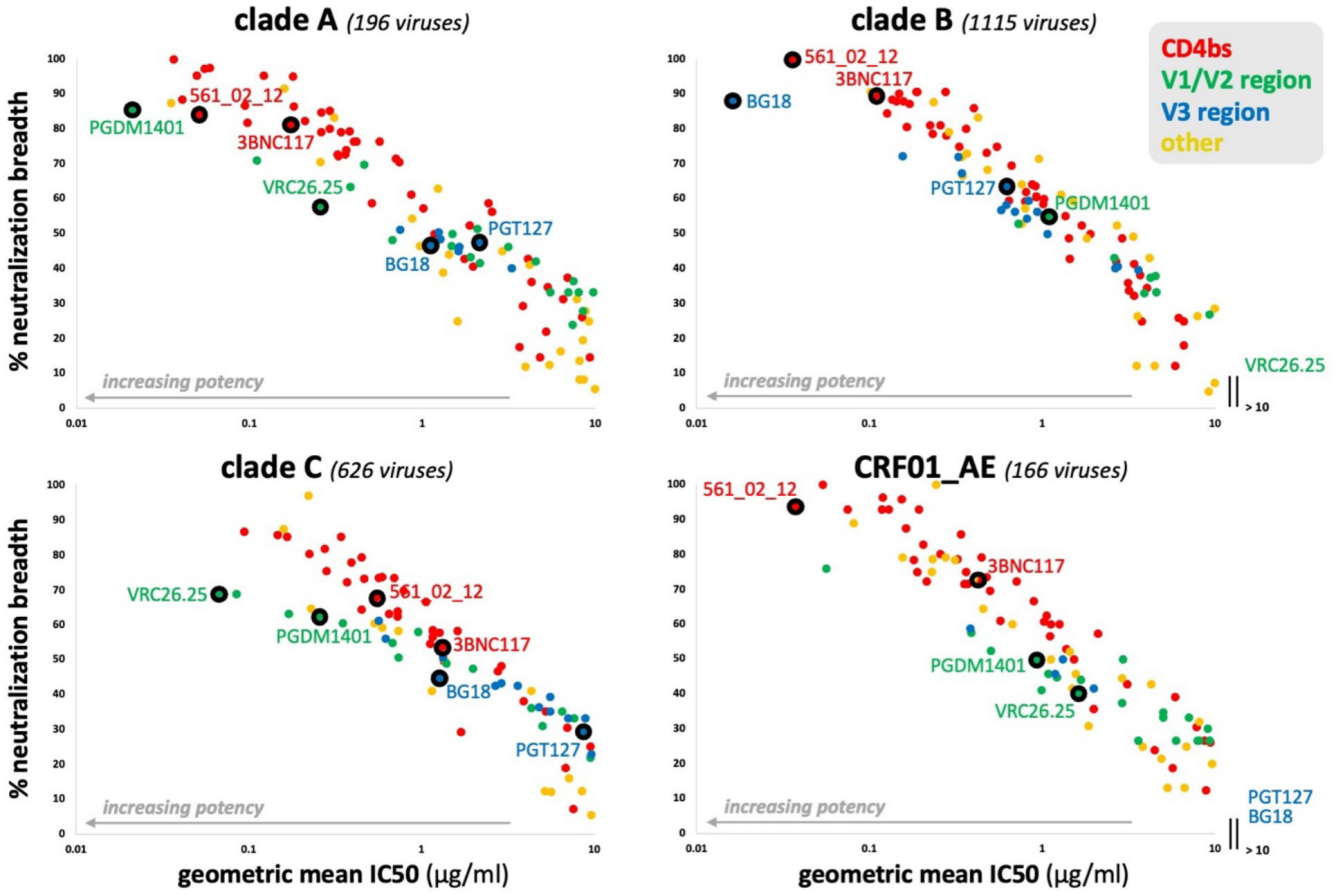
Antibody potency versus breadth for HIV-1 clades A, B, C, and CRF01_AE. CAByN default settings were used except that only one clade was analyzed at a time. All antibodies with a geometric mean IC50 ≤ 10 µg/ml, not just those meeting the criteria set by CAByN, were plotted. Only antibodies with sufficient data to analyze for the following clades were included in the plots: A, B, C, and CRF01_AE. The number of viruses meeting criteria for analysis are listed for each clade.

**Figure 5:**
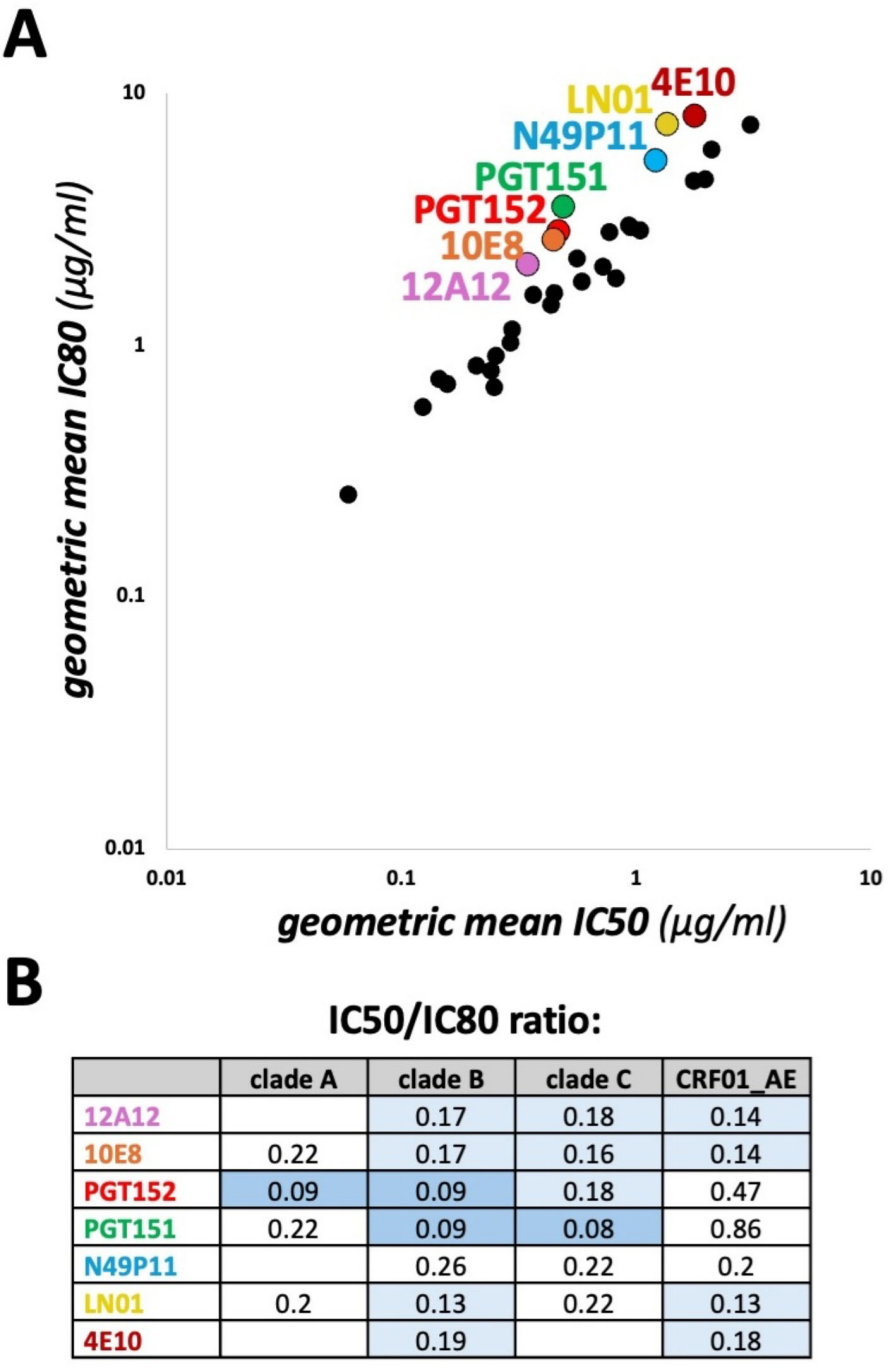
Comparison of geometric mean IC50 vs IC80 values. **(A)** CAByN default settings were used except that the 118 multi-clade panel was selected, and a minimum of 100 viruses was chosen. This minimum number of viruses was chosen to ensure that similar viruses were tested for both the geometric mean IC50 and IC80 calculations. All antibodies with a geometric mean IC50 ≤ 10 µg/ml, not just those meeting the criteria set by CAByN, were plotted. **(B)** Geometric mean IC50/IC80 ratios for select clades and antibodies are listed. CAByN default settings were used except that the 118 multi-clade panel was selected, and the following minimum number of viruses were chosen for clades A, B, C, and CRF01_AE, respectively: 16, 21, 34, and 11. Minimum number of viruses were chosen to ensure that similar viruses were tested for both the geometric mean IC50 and IC80 calculations. Empty cells indicate insufficient data to calculate.

Data used for the 10–1074 example in the “Method” section above was retrieved June 4, 2025. Data used for **Table 1** was retrieved May 5, 2025. The screenshot used for **Figure 2** was taken May 5, 2025. Data used for **Figures 3**–**5** was retrieved May 1, 2025.

## 3 Results

### 3.1 Development of our CAByN tool

Our group, the Los Alamos HIV Databases, was interested in helping HIV researchers identify bnAbs but recognized that researchers likely valued proposed bnAb criteria differently. Therefore, we have developed a web-based tool, Choose Antibodies by Neutralization (CAByN), allowing users to modify several potential criteria for bnAbs (**Figure 2**). Data analyzed by CAByN comes from our CATNAP database (11). CAByN allows users to select IC50 or IC80 data to analyze. A minimum number of viruses analyzed can be set to ensure that minimally assayed antibodies are not returned. CAByN also allows users to set numerical cutoffs for the following criteria: potency threshold for neutralization in a single assay, geometric mean IC50 or IC80 threshold across all assays selected, number of clades/CRFs neutralized, and neutralization breadth.

Additionally, CAByN allows users to restrict analyses to specific viral clades/circulating recombinant forms (CRFs), as well as limit analysis to certain viral panels (e.g. 118 multi-clade panel) or tier 2/3 viruses. Specific antibody binding regions (e.g. CD4bs) can be chosen, and results can be restricted to natural monoclonal antibodies.

### 3.2 CAByN default settings

CAByN’s default settings were carefully chosen to provide users with a list of antibodies that, if not bnAbs by all definitions, still have unusual potency or breadth. Analyzing IC50 data is selected by default, as CATNAP’s database currently contains more IC50 data than IC80 data. All numerical default settings were chosen assuming that IC50 is selected; if IC80 is selected, these values likely will need to be adjusted. The IC50 threshold for neutralization in a single assay is 1 µg/ml, which has been used as a threshold previously (12). The geometric mean IC50 across assays and minimum neutralization breadth were set to 3.6 µg/ml and 30%, respectively, based on criteria proposed for bnAbs (10). Minimum number of clades/CRFs neutralized was initially set to 7 but was later adjusted to 1 based on concerns that viral subtype does not define neutralization activity (13) but instead access to epitopes in Env does. However, differential glycosylation across strains is apparent (14) and loss of some glycosylation sites appears to occur in a strain-dependent manner (15). CAByN also sets a minimum of 12 viruses needing to be tested as a default. This is to minimize the possibility of CAByN returning antibodies meeting set criteria with little supporting data. Also, if it can be assumed viruses tested represent viral diversity, a 12-virus panel appears to assess neutralization activity as well as larger panels (16).

By default, CAByN will analyze all HIV-1 viruses in CATNAP, though it is possible to restrict to certain viral subtypes or CRFs (subtypes A, B, C, D, F, G, CRF01_AE, CRF02_AG, CRF07_BC, all M group viruses, other M group viruses not selectable, and N/O/P). Viruses assessed can also be restricted to tier 2/3 viruses, or to specific viral panels (currently, we offer from CATNAP the 118 and 208 multi-clade panels, the AMP panel, the most common 200 viruses panel, and all CATNAP panel viruses). By default, only natural monoclonal antibodies (as opposed to polyclonal antibodies, engineered monoclonals, or other antibody types) are returned. It is also possible to limit results to certain antibody binding regions (**Figure 1**) (CD4bs, CD4i, V1/V2, V3, gp41 MPER, gp120–41 interface, gp120 silent face, and fusion peptide), although all regions are returned by default.

CAByN’s default settings will return antibodies with potent neutralization activity across multiple viruses. In general, small adjustments to any numerical criteria in CAByN do not dramatically alter the number of antibodies returned, suggesting that, together, the default criteria impose a high level of stringency upon what antibodies are determined to be the top neutralizing antibodies in each search (**Figure 3A**). In general, there is a strong association between antibody potency and breadth across antibodies in CATNAP (**Figure 3B**). Increasing CAByN’s IC50 threshold (such that less potent antibodies are included in the analysis) returns antibodies with increasingly lower neutralization breadth, and increasing the threshold for neutralization breadth returns antibodies with increasingly higher potency (lower IC50 values).

### 3.3 Insights from CAByN searches

CAByN searches, each limited to a single clade/CRF but otherwise using CAByN’s default settings, were performed. Antibody potency and breadth were compared across clades A, B, C, and CRF01_AE, and while many antibodies had relatively similar breadth and potency across these subtypes (e.g. CD4 binding site-directed antibodies 561_02_12 and 3BNC117), other antibodies had limited activity against certain clades (e.g. VRC26.25 against clade B viruses and most V3-directed antibodies against CRF01_AE). Additionally, for many other antibodies, antibody breadth and potency varied substantially across clades (**Figure 4**).

We also used CAByN to assess the correlation between geometric mean IC50 and IC80 values. We observed that, for several antibodies, the ratio of IC50 to IC80 values was lower than expected (**Figure 5A**). The antibody with the lowest ratio, PGT151, has previously been described as having incomplete neutralization (17), a phenomenon often attributed to incomplete glycosylation. Incomplete neutralization has also been reported for 10E8, PGT152, and 4E10 (18–20). For certain antibodies, this ratio also varied by viral clade (**Figure 5B**). Further investigation of amino acid residues associated with incomplete neutralization, including potential glycosylation sites, could be accomplished using our CATNAP database.

## 4 Discussion

Our new tool, CAByN, facilitates nuanced searches for neutralizing antibodies in LANL’s HIV Databases’ CATNAP antibody neutralization database (11). CAByN allows users to specify the following numerical criteria for analysis of IC50 or IC80 data: potency threshold for neutralization in a single assay, geometric mean IC50 or IC80 threshold across all assays selected, number of clades/CRFs neutralized, and neutralization breadth. CAByN also allows users to restrict analyses to certain viral clades or panels, or certain antibody types (natural monoclonal antibodies) or antibody binding regions. CAByN returns a list of antibodies meeting users’ criteria, as well as downloadable files of antibodies meeting criteria and all antibodies assessed. Users also have the option of using default criteria to generate a list of bnAbs/bnAb-like antibodies. We envision CAByN being used to identify antibodies of interest for further analysis using tools in our databases such as CombiNAber (21) or CATNAP.

## Data Availability

The raw data supporting the conclusions of this article will be made available by the authors, without undue reservation.

## References

[1] McMichael AJ Borrow P Tomaras GD Goonetilleke N Haynes BF. The immune response during acute HIV-1 infection: clues for vaccine development. Nat Rev Immunol. (2010) 10:11–23. doi: 10.1038/nri2674, PMID: 20010788 PMC3119211

[2] Simek MD Rida W Priddy FH Pung P Carrow E Laufer DS. Human immunodeficiency virus type 1 elite neutralizers: individuals with broad and potent neutralizing activity identified by using a high-throughput neutralization assay together with an analytical selection algorithm. J Virol. (2009) 83:7337–48. doi: 10.1128/JVI.00110-09, PMID: 19439467 PMC2704778

[3] Kwon YD Chuang GY Zhang B Bailer RT Doria-Rose NA Gindin TS. Surface-matrix screening identifies semi-specific interactions that improve potency of a near pan-reactive HIV-1-neutralizing antibody. Cell Rep. (2018) 22:1798–809. doi: 10.1016/j.celrep.2018.01.023, PMID: 29444432 PMC5889116

[4] Sprumont A Rodrigues A McGowan SJ Bannard C Bannard O. Germinal centers output clonally diverse plasma cell populations expressing high- and low-affinity antibodies. Cell. (2023) 186:5486–5499.e13. doi: 10.1016/j.cell.2023.10.022, PMID: 37951212 PMC7617393

[5] Guttman M Cupo A Julien JP Sanders RW Wilson IA Moore JP. Antibody potency relates to the ability to recognize the closed, pre-fusion form of HIV Env. Nat Commun. (2015) 6:6144. doi: 10.1038/ncomms7144, PMID: 25652336 PMC4338595

[6] Mayer BT deCamp AC Huang Y Schiffer JT Gottardo R Gilbert PB. Optimizing clinical dosing of combination broadly neutralizing antibodies for HIV prevention. PloS Comput Biol. (2022) 18:e1010003. doi: 10.1371/journal.pcbi.1010003, PMID: 35385469 PMC9084525

[7] Powell RL Kinge T Nyambi PN. Infection by discordant strains of HIV-1 markedly enhances the neutralizing antibody response against heterologous virus. J Virol. (2010) 84:9415–26. doi: 10.1128/JVI.02732-09, PMID: 20631143 PMC2937625

[8] Govindan R Stephenson KE. HIV vaccine development at a crossroads: new B and T cell approaches. Vaccines (Basel). (2024) 12:1043. doi: 10.3390/vaccines12091043, PMID: 39340073 PMC11435826

[9] Montefiori DC Roederer M Morris L Seaman MS. Neutralization tiers of HIV-1. Curr Opin HIV AIDS. (2018) 13:128–36. doi: 10.1097/COH.0000000000000442, PMID: 29266013 PMC5802254

[10] Griffith SA McCoy LE. To bnAb or Not to bnAb: Defining Broadly Neutralizing Antibodies Against HIV-1. Front Immunol. (2021) 12:708227. doi: 10.3389/fimmu.2021.708227, PMID: 34737737 PMC8560739

[11] Yoon H Macke J West AP Jr Foley B Bjorkman PJ Korber B. CATNAP: a tool to compile, analyze and tally neutralizing antibody panels. Nucleic Acids Res. (2015) 43:W213–9. doi: 10.1093/nar/gkv404, PMID: 26044712 PMC4489231

[12] Wu X Yang ZY Li Y Hogerkorp CM Schief WR Seaman MS. Rational design of envelope identifies broadly neutralizing human monoclonal antibodies to HIV-1. Science. (2010) 329:856–61. doi: 10.1126/science.1187659, PMID: 20616233 PMC2965066

[13] Moore JP Cao Y Leu J Qin L Korber B Ho DD. Inter- and intraclade neutralization of human immunodeficiency virus type 1: genetic clades do not correspond to neutralization serotypes but partially correspond to gp120 antigenic serotypes. J Virol. (1996) 70:427–44. doi: 10.1128/JVI.70.1.427-444.1996, PMID: 8523556 PMC189832

[14] Stewart-Jones GB Soto C Lemmin T Chuang GY Druz A Kong R. Trimeric HIV-1-env structures define glycan shields from clades A, B and G. Cell. (2016) 165:813–26. doi: 10.1016/j.cell.2016.04.010, PMID: 27114034 PMC5543418

[15] Wang W Nie J Prochnow C Truong C Jia Z Wang S. A systematic study of the N-glycosylation sites of HIV-1 envelope protein on infectivity and antibody-mediated neutralization. Retrovirology. (2013) 10:14. doi: 10.1186/1742-4690-10-14, PMID: 23384254 PMC3648360

[16] deCamp A Hraber P Bailer RT Seaman MS Ochsenbauer C Kappes J. Global panel of HIV-1 Env reference strains for standardized assessments of vaccine-elicited neutralizing antibodies. J Virol. (2014) 88:2489–507. doi: 10.1128/JVI.02853-13, PMID: 24352443 PMC3958090

[17] Falkowska E Le KM Ramos A Doores KJ Lee JH Blattner C. Broadly neutralizing HIV antibodies define a glycan-dependent epitope on the prefusion conformation of gp41 on cleaved envelope trimers. Immunity. (2014) 40:657–68. doi: 10.1016/j.immuni.2014.04.009, PMID: 24768347 PMC4070425

[18] Kim AS Leaman DP Zwick MB. Antibody to gp41 MPER alters functional properties of HIV-1 Env without complete neutralization. PloS Pathog. (2014) 10:e1004271. doi: 10.1371/journal.ppat.1004271, PMID: 25058619 PMC4110039

[19] McCoy LE Falkowska E Doores KJ Le K Sok D van Gils MJ. Incomplete neutralization and deviation from sigmoidal neutralization curves for HIV broadly neutralizing monoclonal antibodies. PloS Pathog. (2015) 11:e1005110. doi: 10.1371/journal.ppat.1005110, PMID: 26267277 PMC4534392

[20] Li H Zony C Chen P Chen BK. Reduced potency and incomplete neutralization of broadly neutralizing antibodies against cell-to-cell transmission of HIV-1 with transmitted founder envs. J Virol. (2017) 91:e02425–16. doi: 10.1128/JVI.02425-16, PMID: 28148796 PMC5391450

[21] Wagh K Bhattacharya T Williamson C Robles A Bayne M Garrity J. Optimal combinations of broadly neutralizing antibodies for prevention and treatment of HIV-1 clade C infection. PloS Pathog. (2016) 12:e1005520. doi: 10.1371/journal.ppat.1005520, PMID: 27028935 PMC4814126

[22] Wyatt R Kwong PD Desjardins E Sweet RW Robinson J Hendrickson WA. The antigenic structure of the HIV gp120 envelope glycoprotein. Nature. (1998) 393:705–11. doi: 10.1038/31514, PMID: 9641684

